# Diversity of Chemical Structures and Biosynthesis of Polyphenols in Nut-Bearing Species

**DOI:** 10.3389/fpls.2021.642581

**Published:** 2021-04-06

**Authors:** Chaiwat Aneklaphakij, Tomoki Saigo, Mutsumi Watanabe, Thomas Naake, Alisdair R. Fernie, Somnuk Bunsupa, Veena Satitpatipan, Takayuki Tohge

**Affiliations:** ^1^Department of Pharmacognosy, Faculty of Pharmacy, Mahidol University, Bangkok, Thailand; ^2^Graduate School of Biological Science, Nara Institute of Science and Technology, Ikoma, Japan; ^3^Max-Planck-Institute of Molecular Plant Physiology, Potsdam, Germany

**Keywords:** flavonoids, chemical diversity, nuts, comparative genomics, polyphenols, health benefits

## Abstract

Nuts, such as peanut, almond, and chestnut, are valuable food crops for humans being important sources of fatty acids, vitamins, minerals, and polyphenols. Polyphenols, such as flavonoids, stilbenoids, and hydroxycinnamates, represent a group of plant-specialized (secondary) metabolites which are characterized as health-beneficial antioxidants within the human diet as well as physiological stress protectants within the plant. In food chemistry research, a multitude of polyphenols contained in culinary nuts have been studied leading to the identification of their chemical properties and bioactivities. Although functional elucidation of the biosynthetic genes of polyphenols in nut species is crucially important for crop improvement in the creation of higher-quality nuts and stress-tolerant cultivars, the chemical diversity of nut polyphenols and the key biosynthetic genes responsible for their production are still largely uncharacterized. However, current technical advances in whole-genome sequencing have facilitated that nut plant species became model plants for omics-based approaches. Here, we review the chemical diversity of seed polyphenols in majorly consumed nut species coupled to insights into their biological activities. Furthermore, we present an example of the annotation of key genes involved in polyphenolic biosynthesis in peanut using comparative genomics as a case study outlining how we are approaching omics-based approaches of the nut plant species.

## Introduction

Nuts, such as chestnut and hazelnut, are oil-rich seeds comprising of an edible fruit with a hard outer shell attached to a cupule. Additionally, drupe seeds such as almond, peanut, pistachio, walnut, macadamia, pecan, and cashew nut also contain a hard shell and are thus referred to as “nuts.” Since nuts contain precious types of phytonutrients which exhibit beneficial health-promoting properties, they are regarded as one of the most valuable culinary crops (Ros, 2010; Bodoira and Maestri, 2020). Indeed, consuming nuts provides a rich source of nutritional components, including fatty acids, minerals, vitamins, proteins, and fibers (Ros, 2010; Chen C. Y. O. et al., 2019). In addition, nuts were also found as a rich source of “plant-specialized (secondary) metabolites” which are a vast array of bioactive compounds. Plant-specialized compounds tend to act as stress protectants against biotic and abiotic stresses from the external environment (Tohge et al., 2013b, 2016; Kessler and Kalske, 2018; Pais et al., 2018; Scossa et al., 2019). These metabolites broadly correspond to important physiological and ecological functions, for example, attracting insects for pollination by volatiles and color; as antifeedants and allelochemicals against herbivores; for visible pigmentation; and for lightening from stress conditions, e.g., ultraviolet radiation, elicitors, temperature, and water deficiency (Bodoira and Maestri, 2020; Corso et al., 2020; Yuan and Grotewold, 2020).

**FIGURE 1:**
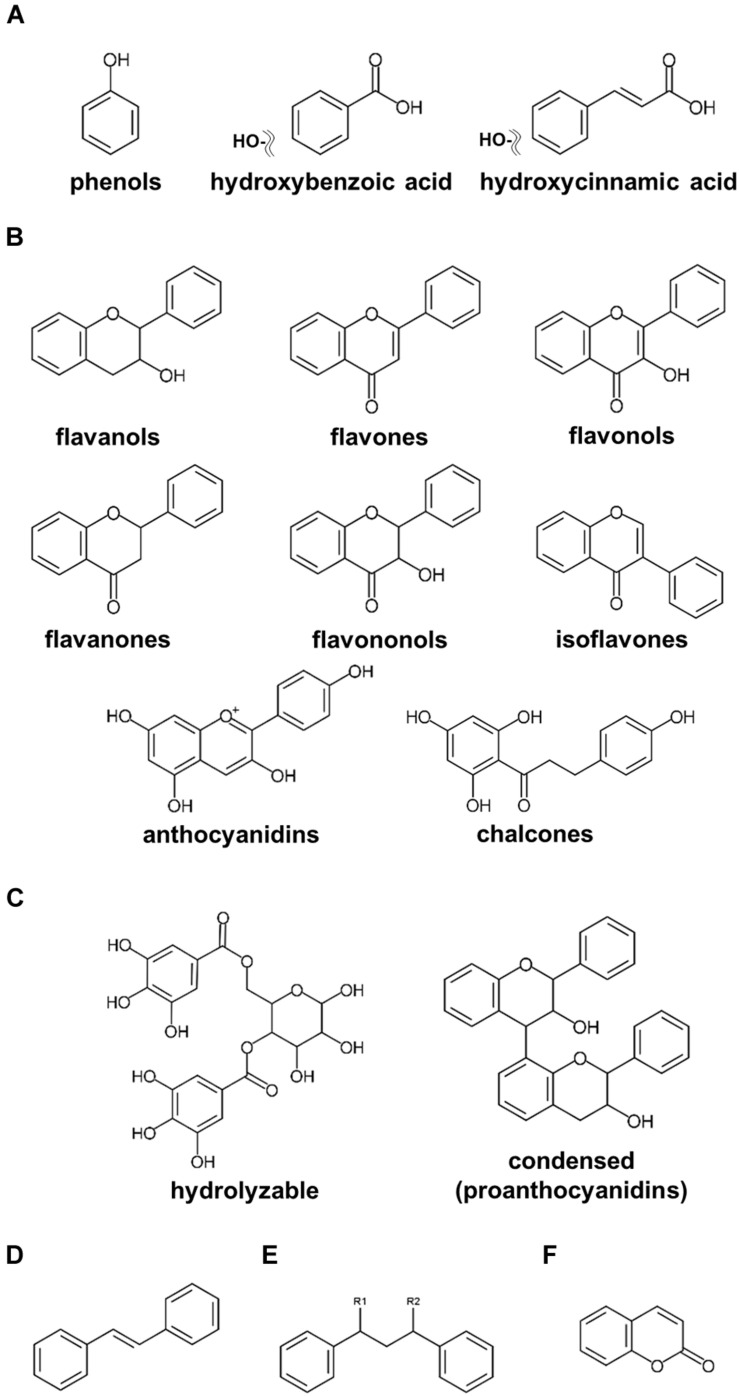
Core structures of polyphenolics found in major nut plant species. **(A)** phenols and phenolic acids. **(B)** flavonoids. **(C)** tannins. **(D)** stilbenoids. **(E)** lignans. **(F)** coumarins.

Plant-specialized metabolites are allocated to three main categories, namely, terpenoids, alkaloids, and polyphenols, classified on their chemical core skeletons and biosynthetic pathways (Tohge et al.,2013a,b, 2014; Chen C. Y. O. et al., 2019; Bodoira and Maestri, 2020; Yuan and Grotewold, 2020). Polyphenols are ubiquitously present among fruits, vegetables, and seeds, including nuts, with multiple claims of these compounds presenting human health beneficial effects on human health (Lepiniec et al., 2006; Tohge and Fernie, 2017; Hano and Tungmunnithum, 2020; Alseekh et al., 2020a). Numerous research publications investigating polyphenols in nuts have been published (Bolling et al., 2011; Alasalvar and Bolling, 2015; Bodoira and Maestri, 2020). According to our current update, polyphenols in nut plant species are classified into six major groups, including phenols, flavonoids, tannins, stilbenoids, lignans, and coumarins (Figure 1). Phenols including phenolic acids (C6–C1 skeleton) and hydroxycinnamic acids (C6–C3) are the compounds containing an aromatic ring attached with at least one hydroxyl moiety (Figure 1A; Bodoira and Maestri, 2020; Hano and Tungmunnithum, 2020). Flavonoids (C6–C3–C6), one of the largest classes of specialized metabolites, are distributed extensively in the plant kingdom (Figure 1B; Tohge et al.,2013a,b; Mathesius, 2018). Flavonoids reported in seeds of ten major nut plant species are subdivided into eight subclasses, i.e., flavanols, flavones, flavonols, flavanones, flavanonols, isoflavones, anthocyanins, and proanthocyanidins (Figures 1B,C; Tohge et al., 2013b; Saigo et al., 2020). Tannins, comprising of hydrolyzable and condensed tannins, are astringent and bitter plant substances being particularly numerous in nuts seeds (Landete, 2011). Stilbene compounds are comprised of two aromatic rings connecting with an ethylene bridge (C6–C2–C6 backbone) and commonly found as monomers and oligomers in both aglycone and glycoside forms (Figure 1D; Akinwumi et al., 2018; Hano and Tungmunnithum, 2020). Oxidative dimerization of two or more phenylpropanoid molecules produces lignans for lignin synthesis, and as such important precursors for the development of plant vessels and cell walls (Figure 1E; Barker, 2019). Coumarins are plant benzo-α-pyrone compounds generated by the reaction of pyran-benzene ring condensation (Figure 1F; Küpeli Akkol et al., 2020).

With respect to biological or pharmaceutical activities, polyphenols have been found to be beneficial components in both human health promotion and disease prevention. They were shown to exhibit antioxidative, anticancer, cardio-protective, antibacterial, anti-inflammatory, and immune system-promoting properties and to exert protection for skin against UV radiation, against neurodegenerative diseases, chronic diseases, obesity, and diabetes, and against the current pandemic coronavirus disease (COVID-19), being reported (Nayak et al., 2015; Tohge and Fernie, 2017; Puksasook et al.,2017a,b; Cory et al., 2018; Tungmunnithum et al., 2018; Renaud and Martinoli, 2019; Ngwa et al., 2020). Several review articles focus on the health benefits of nut consumption (Ros, 2010; Bolling et al., 2011; Alasalvar and Bolling, 2015), with the major groups of polyphenols present in nut seeds being characterized (Bolling et al., 2011; Alasalvar and Bolling, 2015; Bodoira and Maestri, 2020). Currently, the chemical diversity of specialized metabolites and metabolic polymorphisms have specifically highlighted the decoration of polyphenols, with such decorations being found to be a key factor in the enhancement of bioactivity of specialized compounds (Tohge et al., 2016; Peng et al., 2017). In fact, most of the aforementioned biological activities of polyphenols are derived from not only the aglycone form but also the decorated form. The capacity of biological activities such as antioxidant capacity significantly depends on the chemical structures, since these dramatically affect the bioavailability and especially absorption of the compounds (Cipolletti et al., 2018; Gulcin, 2020). Although the core biosynthetic pathways of many polyphenols are conversed among genetically and taxonomically distant plant species, these species often accumulate polyphenols in a tissue-specific manner. In the case of seed-specific specialized metabolites, polyphenols are assumed to be involved in environmental stress protection during seed desiccation and dormancy. As such, the elucidation and understanding of physiological functions of such tissue-specific specialized metabolites are highly valuable.

Current technological and theoretical development of omics-based approaches has enabled that the genome-wide characterization of biosynthetic genes can be carried out, representing an important route by which phenol and polyphenol production could be enhanced in plants (Butelli et al., 2008; Tohge and Fernie, 2010; Zhang et al., 2015; Fernie and Tohge, 2017; Alseekh et al., 2020b). Notably, almost all genes encoding enzymes responsible for structure decoration remain ambiguous. However, as yet, genomic data is only available for peanut and almond; therefore, studies concerning nut plant polyphenolics are not as extensive as they could have been. As such, it is important to update and synthesize the collective information concerning chemical diversity in nut plant species. Given the presence of some species-specific polyphenolics in nuts, it is likely that such a compendium will prove a useful resource for biological activity investigations.

**FIGURE 2:**
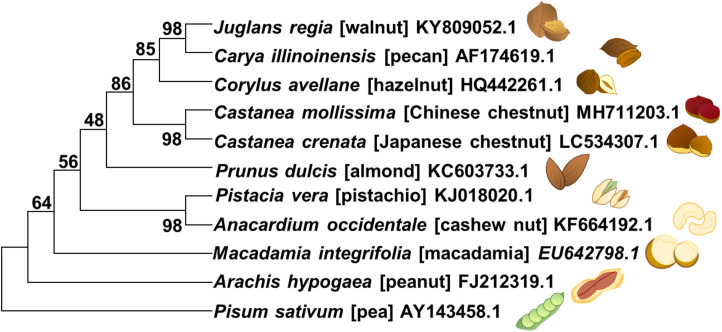
Phylogenetic relationship of ten nut plant species presented in this review. The phylogenetic tree was constructed by MEGA X (Kumar et al., 2018) by using the sequences of ITS (internal transcribed spacer) gene of each species which were retrieved from the NCBI database. *P. sativum* was considered as an outgroup. The parameters for maximum-likelihood analysis were shown as followed: Kimura 2-parameter model, complete deletion, and bootstrap (1000 replicates). Values presented on the branches demonstrated bootstrap support in percentage.

In this review, the current knowledge of polyphenolic compounds in major nut plant species is summarized in terms of their biological activities, chemical diversity, and biosynthetic genes. Ten eminently consumed nut plant species, namely, groundnut/peanut (*Arachis hypogaea*), almond (*Prunus dulcis*), pistachio (*Pistacia vera*), Japanese chestnut (*Castanea crenata*), Chinese chestnut (*Castanea mollissima*), walnut (*Juglans regia*), hazelnut (*Corylus avellana*), macadamia (*Macadamia integrifolia*), pecan (*Carya illinoinensis*), and cashew nut (*Anacardium occidentale*), are presented (Figure 2 and Table 1). We additionally propose a future perspective for generating an integrative omics approach for functional genomics utilizing polyphenolic biosynthesis in nut plant species as a case study.

**TABLE 1:** Nut plant species presented in this article.

Name	Species name	Genome sequencing	BioProject ID (NCBI)
Groundnut/peanut	*Arachis hypogaea*	Bertioli et al., 2019	PRJNA419393,PRJNA480120, PRJNA680825
Almond	*Prunus dulcis*	Sánchez-Pérez et al., 2019	PRJDB7547, PRJNA497779
Pistachio	*Pistacia vera*	Zeng et al., 2019	PRJNA578116
Japanese chestnut	*Castanea crenata*		
Chinese chestnut	*Castanea mollissima*		PRJNA559042
Walnut	*Juglans regia*	Martínez-García et al., 2016	PRJNA350852, PRJNA445704
Hazelnut	*Corylus avellana*	Lucas et al., 2020	PRJEB31933
Macadamia	*Macadamia integrifolia*		
Pecan	*Carya illinoinensis*		
Cashew nut	*Anacardium occidentale*		

## Polyphenolics in Nuts and Their Bioactivities Associated With Health-Promoting Benefits

Several popular nut plant species are consumed as snacks and food supplements, since they are rich in phytonutrients especially fatty acids, protein, minerals, and polyphenolics (Ros, 2010; Vinson and Cai, 2012). To date, research in food chemistry has suggested several biological activities of nut extracts with studies on the antioxidant activity being particularly prominent. From a health beneficial perspective, antioxidants are responsible for the elimination of reactive oxygen species (ROS) or free radical molecules such as superoxide, nitric oxide, and hydrogen peroxide radicals from the human body in order to prevent the generation of hazardous substances which underlie many chronic diseases (Gulcin, 2020). Polyphenols are one of the best-known and major sources of natural antioxidants due to their effective scavenging activity resulting from the presence of several hydroxyl groups present on the structures, especially those on the *ortho-* and *para-* positions of the aromatic ring (Shahidi and Ambigaipalan, 2015; Gulcin, 2020). On the basis of their total polyphenolic quantity, the highest antioxidant activities were found in raw walnut and roasted almond (Vinson and Cai, 2012). The biological activities of nut polyphenol antioxidants against major stress such as oxidative stress, aging, and age-related disease prevention were summarized in previous reviews (Ros, 2010; Bodoira and Maestri, 2020; Hano and Tungmunnithum, 2020). Nut consumption has been suggested to play a key role in cardio-protection by reducing cardiovascular risk factors, including coronary heart disease, hypertension, and blood cholesterol levels (Ros, 2010; De Souza et al., 2017). In a recent study, almond skin extract was found to show antimicrobial and antiviral activities against *Staphylococcus aureus* and herpes simplex virus type I, respectively (Musarra-Pizzo et al., 2019). Due to such indication of nut consumption and health-promoting benefits, polyphenols contained in culinary nuts have been focused and studied with quantification of known health-promoting polyphenols in food chemistry research. Bioactivities of major and specific polyphenolics in nut plant species are summarized in Table 2. Given that health beneficial components such as resveratrols, chlorogenic acids, catechins, and rutin are detected in nuts, health-promoting benefits of culinary nuts are considered with bioactivities and concentrations of these polyphenols. Additionally, nut-specific polyphenolics such as cardanols and anacardic acid were found as cashew nut-specific antioxidant compounds (Table 2). Ellagic acid, which is present in several nuts including almond, walnut, pecan, Japanese chestnut, and hazelnut, has been reported as an inhibitor of inflammatory mediator molecules such as cyclooxygenase and nuclear factor κB, providing anti-inflammatory activity (El-Shitany et al., 2014). Anacardic acid from cashew nut, ellagic acid from walnut and pecan, genistein from peanut and hazelnut, and resveratrol from peanut have demonstrated anticancer properties with numerous molecular targets (Falasca et al., 2014). Resveratrol and its prenylated derivatives in peanut have been reported to mitigate against neurodegenerative diseases such as Alzheimer’s and Parkinson’s disease via their antioxidant, anti-β-amyloid aggregation, anti-β-secretase, neuroprotective, and neuritogenicity properties (Puksasook et al.,2017a,b; Navarro et al., 2018). Captivatingly, myricetin which is found in pistachio and hazelnut as well as resveratrol in peanut, almond, and pistachio are recently claimed as potential phytochemical compounds that could counteract the current COVID-19 pandemic (Han et al., 2020; Ngwa et al., 2020).

**TABLE 2:** Bioactivities of major and specific polyphenolics in nut plant species.

Compound name	Nut species	Bioactivities
**Phenols**		
Cardanols*	Cashew nut	Antioxidant, antimutagenic, and antitumoral activity (Maia et al., 2015; Schneider et al., 2016)
Ellagic acid	Almond, walnut, Japanese chestnut, pecan, hazelnut	An inhibitor of inflammatory mediators (El-Shitany et al., 2014)
Gallic acid	Almond, cashew nut, Chinese chestnut, hazelnut, Japanese chestnut, pecan, pistachio, walnut	Antioxidant, anti-inflammatory, anticancer, antimicrobial, cardiovascular, and gastrointestinal treatment, protective effect on neuropsychological diseases (Zanwar et al., 2014; Kahkeshani et al., 2019)
*p*-Hydroxy benzoic acid	Peanut, almond, walnut, Japanese chestnut, Chinese chestnut, hazelnut, pecan	Osteogenic activity, antimicrobial activity, antifungal, estrogenic, and antimutagenic properties (Cho et al., 1998; Pugazhendhi et al., 2005; Heleno et al., 2015)
Chlorogenic acid	Almond, Chinese chestnut, hazelnut, peanut, pecan, pistachio, walnut	Antioxidant, anti-hepatitis B virus, antidiabetic effect, DNA protective effect, neuroprotective effect, protection from cardiovascular diseases (Sato et al., 2011; Zuo et al., 2015; Gil and Wianowska, 2017)
*p*-Coumaric acid	Peanut, almond, walnut, Japanese chestnut, Chinese chestnut, cashew nut, hazelnut	Antioxidant, hyperlipidemia treatment, antimicrobial, antiviral, anti-inflammatory, anticancer, antidiabetic (Lee et al., 2019; Shen et al., 2019)
Anacardic acid and its derivatives*	Cashew nut	Antioxidant, antibacterial, cytotoxicity against *A. salina*, acetylcholinesterase inhibition (Muroi and Kubo, 1996; Maia et al., 2015; Morais et al., 2017)
**Stilbenoids**		
Resveratrol	Peanut, almond, pistachio	Antioxidant,cancer chemopreventive, anti-β-amyloid aggregation, anti-β-secretase activity, neuroprotective, neuritogenicity, cardiovascular protective, anti-inflammatory, blood glucose-lowering, anticancer, anti-obesity (Kuršvietienė et al., 2016; Puksasook et al.,2017a,b)
**Flavonoids: flavonol**		
(+)-Catechin	Peanut, almond, pistachio, walnut, pecan, Chinese chestnut, cashew nut, hazelnut	Antioxidant, antimicrobial, antiviral, anti-inflammatory, anti-allergenic, anticancer, prevention of cardiovascular diseases, and neurodegenerative diseases (Isemura, 2019; Bae et al., 2020)
(–)-Epicatechin	Peanut, almond, pistachio, pecan, cashew nut, hazelnut, walnut	Antioxidant, antidiabetes, anticancer, anti-inflammatory, antihypertensive, antidyslipidemic (Abdulkhaleq et al., 2017; Bernatova, 2018)
Flavonoids: flavone		
Luteolin	Peanut, almond, pistachio, Chinese chestnut	Antioxidant, cardioprotective effects, anti-inflammatory, antidiabetic, antimicrobial, anticancer (Lin et al., 2008; Dong et al., 2017; Luo et al., 2017)
**Flavonoids: flavonol**		
Quercetin	Peanut, almond, pistachio, Chinese chestnut, hazelnut, walnut	Antioxidant, anti-inflammatory, cardiovascular disease prevention, neurodegenerative disorders treatment, anticancer, antibacterial, antiviral (Anand David et al., 2016; Xu et al., 2019)
Rutin	Almond, pistachio, walnut, Chinese chestnut, hazelnut	Antioxidant, neuroprotective, hepatoprotective, cardioprotective, antifungal, antimalarial, antibacterial, anticancer (Gao et al., 2002; Ganeshpurkar and Saluja, 2017)
Isoquercitrin	Almond, pistachio, walnut, hazelnut	Antioxidant, neurological disorders, anti-allergic, antidiabetic, anti-inflammatory (Palazzolo et al., 2012; Valentová et al., 2014)
**Flavonoids: flavanone**		
Eriodictyol	Peanut, almond, pistachio, Chinese chestnut, hazelnut	Antioxidant, cardioprotective, skin protection, antitumor, antidiabetic, anti-inflammatory, cytoprotective, hepatoprotective, neuroprotective (Deng et al., 2020; Li et al., 2020)
**Lignans**		
(+)-Lariciresinol	Almond, cashew nut, chestnut, hazelnut, peanut, pecan, pistachio, walnut	Antifungal, antibacterial (Hwang et al., 2011; Bajpai et al., 2017)
(–)-Matairesinol	Almond, cashew nut, chestnut, hazelnut, peanut, pecan, pistachio, walnut	Antioxidant, anti-osteoclastogenic, anti-angiogenic, anticancer, antifungal, IgE-suppressive activity (Yamauchi et al., 2006; Kawahara et al., 2010; Choi et al., 2014)

*Specific present in cashew nut.

## Chemical Structural Diversity of Polyphenolics Among Seeds of Nut Plant Species

**FIGURE 3:**
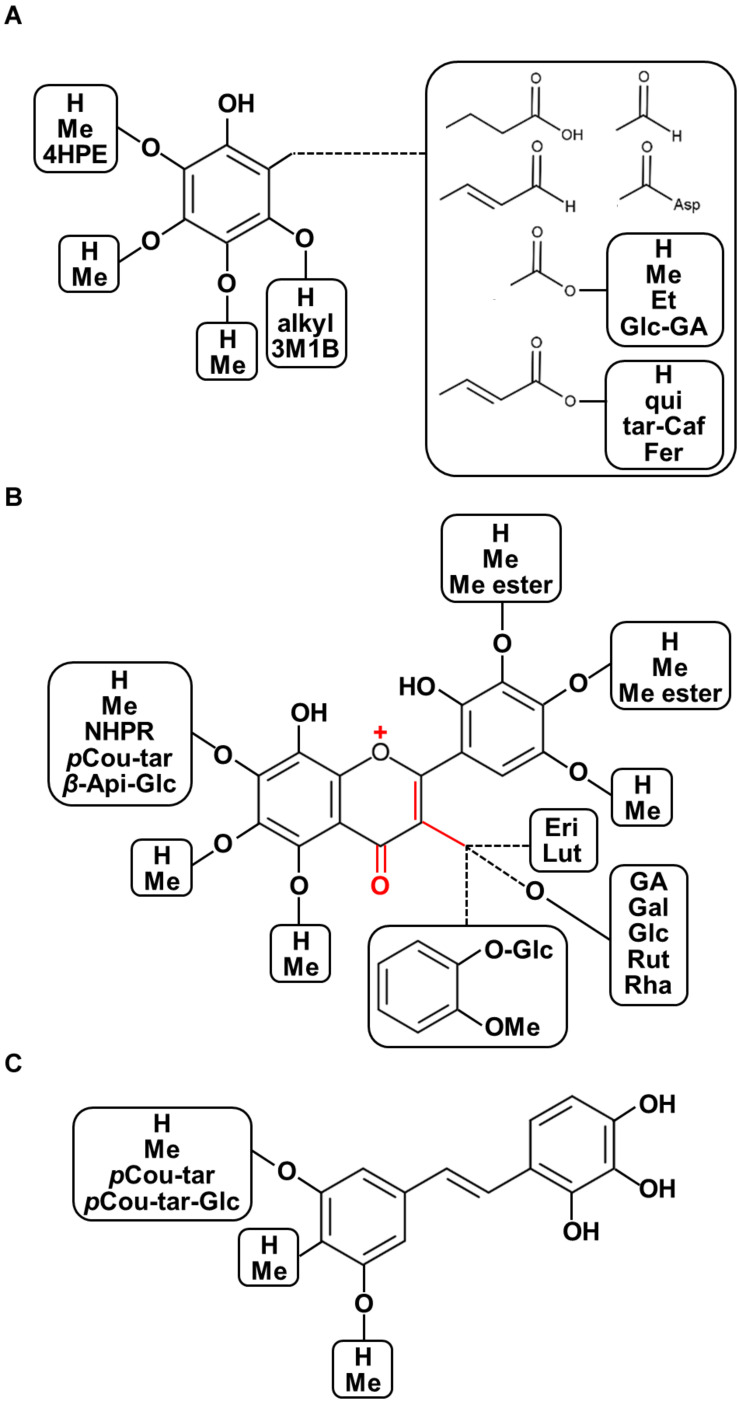
Diversity of chemical structures of polyphenolics found in seeds of nut plant species. **(A)** Phenols and phenolic acid derivatives, **(B)** flavonoids, and **(C)** stilbenoids. Abbreviations: OMe, methoxy; 4HPE, 4-(hydroxypheny)ethylene; Asp, aspartate; Me, methyl; Et, ethyl; qui, quinic acid; tar, tartaric acid; fer, ferulic acid; 3M1B, 3-methyl-1-butenyl; *p*Cou-tar, *p*-coumaroyl-tartarate; *p*Cou-tar-Glc, *p*-coumaroyl-tartarate-glucoside; Pre, isoprenyl group; Me ester, methyl ester; NHPR, neohesperidose; Api-Glc, -apiosyl-glucose; Eri, eriodictyol; Lut, luteolin; GA, gallic acid; Gal, galactose; Glc, glucose; Rut, rutinose; and Rha, rhamnose.

The chemo-diversity of plant metabolism is a highly important factor affecting plant ecological processes and plant metabolic evolution (Kessler and Kalske, 2018). Furthermore, the various characteristics of phytochemical structures show diverse modes of action with regard to the prevention and treatment of human diseases given differences in their physicochemical properties. Recent overviews focusing on plant structural diversity in anthocyanins/proanthocyanidins (Saigo et al., 2020), glucosinolates (Blažević et al., 2020), and diterpenoid alkaloids (Shen et al., 2020) have been published. With this regard, for example, some decorations such as oxidation for enhancing acidity, methylation and acylation for reducing polarity, and glycosylation for stability and solubility are considered as the potential factors corresponding to diversification of biological functions. Moreover, the updated plant chemo-diversity database is a powerful tool for enthusing new pharmaceutical drug discovery (Lautié et al., 2020). To illustrate the diversity of chemical structures of polyphenolics found in seeds of nut plant species, both raw and processed nut seeds were included in our chemical diversity analysis from the renowned literature-based phytochemical database KNApSAcK (http://kanaya.naist.jp/KNApSAcK/, searched by plant scientific names in July, 2020; Afendi et al., 2011). Furthermore, several current phytochemical reports were included to illustrate the structural diversity of nut polyphenols. A list of all 214 polyphenols is provided in Supplementary Table 1, while the structural diversity of nut polyphenolic compounds is presented in Figure 3.

### Phenols and Phenolic Acids

Several types of phenols have been found in nut plant species (Table 2 and Figure 3A). Four chemical isomers, *alpha*-, *beta*-, *gamma*-, and *delta*- of tocopherol, were detected in pistachio kernel, walnut kernel, and whole cashew nut (Horvath et al., 2006; Ballistreri et al., 2009; Trox et al., 2011). Cardanols and their derivatives were reported only in the kernel of the cashew nut (Trevisan et al., 2006; Alvarenga et al., 2016; Bodoira and Maestri, 2020). In pecan and walnut kernel, many glycosylated and methylated ellagic acid derivatives are present at high abundance (De La Rosa et al., 2011; Grace et al., 2014; Regueiro et al., 2014; Robbins et al., 2014; Jia et al., 2018). Gallic, *p*-coumaric, chlorogenic, and *p*-hydroxybenzoic acids are the most abundant phenolic acids found in the kernel and skin of seeds of ten nut plant species. In addition, several phenolic acids were specifically presented in some of the nut plant species. Anacardic acid and derivatives were present in cashew nut and pistachio kernel (Trevisan et al., 2006; Alvarenga et al., 2016; Bodoira et al., 2019; Salehi et al., 2019; Bodoira and Maestri, 2020). Coutaric and fertaric acids are constituents in peanut and hazelnut skin (Ma et al., 2014; Pelvan et al., 2018). Chinese chestnut skin contains high levels of gentisic acid and 2,3,4-trihydroxybenzoic acid (Xu et al., 2020). In fact, Bodoira and Maestri have mentioned that phenolic acids of peanut are found only in the skin (Bodoira and Maestri, 2020). Nevertheless, nut phenolic acids, such as phloretic acid and dihydroxybenzoic acid, were found not only in the skin but also in the kernel of peanut (Bisby, 1994). Apart from common phenolic acids, numerous derivatives of gallic, hydroxybenzoic, and hydroxycinnamic acids, such as esterification with other phenolic acids, hydroxylated and methylated derivatives, are also conspicuously reported in nut seeds (Bodoira and Maestri, 2020). Polymerization of tartaric acid with two other molecules of phenolic acids is produced only in peanut skin reported by Ma et al. (2014).

### Flavonoids

A multitude of flavonoids including flavanols, flavones, flavonols, flavanones, flavanonols, isoflavones, anthocyanins, and proanthocyanidins have been found nut plant species (Table 2 and Figures 1B, 3B). When the hydroxyl group is connected to carbon position three of the C ring, the molecule belongs to the flavanol subclass. Flavonols represent the principal subgroups of flavonoids since they display a rich diversity of derivatives and are also a basic structure of proanthocyanidins (Tohge et al., 2017; Saigo et al., 2020). Catechin and epicatechin are visibly rich in seeds of almost all of the ten major nut plant species. Moreover, their derivatives via esterification with gallic acid, epicatechin-gallate, gallocatechin-gallate, epigallocatechin, and epigallocatechin gallate, were also determined to be abundant in these species being found in whole almond seed (Bolling, 2017), kernel and skin of cashew nut (Salehi et al., 2019), pecan, walnut, Chinese chestnut kernel (Regueiro et al., 2014; Jia et al., 2018; Zhang Y. et al., 2020), and hazelnut skin (Del Rio et al., 2011; Pelvan et al., 2018). Luteolin and apigenin are typical flavones found in peanut skin (Bodoira et al., 2017), almond kernel (Čolić et al., 2017), kernel and the skin of pistachio (Tomaino et al., 2010; Fabani et al., 2013), Chinese chestnut kernel (Zhang Y. et al., 2020), and Japanese chestnut skin (Tuyen et al., 2017). Predominantly among studied nut plant species, whole almond seeds comprise the most diverse types of flavonols, including quercetin, kaempferol, isorhamnetin, and their *O*-glycoside derivatives (Milbury et al., 2006; Monagas et al., 2007; Bolling et al., 2010; Valdés et al., 2015; Bolling, 2017; Čolić et al., 2017). Flavonol derivatives are frequently glycosylated at the hydroxyl group at position three. Aside from major flavonols, in Chinese and Japanese chestnut kernel, minor flavonols such as rhamnetin and morin have been detected (Tuyen et al., 2017; Zhang Y. et al., 2020). Metabolites containing the saturated C ring belong to the flavanone subgroup of flavonoids. Eriodictyol is the main flavanone detected among nut seeds. As for flavonols, several types of flavanones and their derivatives are characterized in whole almond seeds (Milbury et al., 2006; Čolić et al., 2017; Bodoira and Maestri, 2020) with glycosylated derivatives generally displaying glycosylation on the hydroxyl group at position seven. Particularly, naringin (naringenin-7-*O*-neohesperidoside) is a major flavanone derivative in kernels of almond, walnut, and Chinese chestnut as well as in pistachio kernel and skin (Tomaino et al., 2010; Čolić et al., 2017; Vu et al., 2018; Zhang Y. et al., 2020). By contrast, flavanonols and hydroxylated derivatives of flavanones are only minor constituents of nuts; for example, aromadendrin and taxifolin are reported in whole almond seeds (Monagas et al., 2007; Bolling, 2017; Vu et al., 2018). The core structure of isoflavones differs from other flavonoids by linkage of the phenyl ring to position three of ring C supplemented with the ketone group at position four. Isoflavones are not broadly found in nut seeds. However, genistein and its glucosides, daidzein, and daidzin, are present in peanut kernel and skin, whole almond, and pistachio kernel (Ballistreri et al., 2009; Tomaino et al., 2010; Bolling, 2017; Bodoira and Maestri, 2020). Anthocyanidins (aglycone form) and anthocyanins (glycoside form) are extensively known as plant pigments in seeds, flowers, and fruits (Saigo et al., 2020). Cyanidin and its glycoside derivatives, i.e., glucoside and galactoside, were reported as constituents in whole almond seeds (Bolling, 2017) and pistachio kernel and skin (Ballistreri et al., 2009; Tomaino et al., 2010; Fabani et al., 2013; Bodoira and Maestri, 2020). Chalcones or the so-called open-chain flavonoids are a very unique flavonoid subclass in the plant kingdom. Phloretin and its glucoside derivative, phlorizin, are the two chalcones found in the almond kernel and hazelnut skin (Del Rio et al., 2011; Čolić et al., 2017).

### Tannins

Both tannin subgroups, i.e., hydrolyzable and condensed tannins, are abundant in peanut skin, whole almond, and walnut kernel. Hydrolyzable tannins are further allocated into two subgroups gallotannins and ellagitannins (Soares et al., 2020). The latter including strictinin, pedunculagin, tellimagrandin, glasrin, rugosin, casuarinin, and praecoxin (Fukuda et al., 2003; Grace et al., 2014; Regueiro et al., 2014; Robbins et al., 2014; Jia et al., 2018) are more prominent than the former. Brown or non-visible colors of plant seed, peel, and bark are caused by proanthocyanidins or condensed tannins (Saigo et al., 2020). As mentioned above, proanthocyanidin is synthesized by combining flavonol molecules, i.e., catechin, epicatechin, or their galloylated derivatives; polymerization started from dimers by several types of inter flavan linkage (Soares et al., 2020). Proanthocyanidins have been characterized in seed testa of nut plant species, especially in peanut, almond, pistachio, and hazelnut (Lou et al., 1999, 2004; Yu et al., 2006; Monagas et al., 2007; Del Rio et al., 2011; Sarnoski et al., 2012; Fabani et al., 2013; Grace et al., 2014; Slatnar et al., 2015; Bodoira et al., 2017, 2019; Bolling, 2017; Pelvan et al., 2018; Bodoira and Maestri, 2020). As stated in the current review article (Saigo et al., 2020), proanthocyanidins and tannins are arduous to study, since their condensed structures are very complex due to high molecular weight and various types of chemical bond configuration.

### Stilbenoids

Typically, stilbene compounds are rarely found in the seeds of nut plant species. Resveratrol, one of the most well-known stilbenes ubiquitously found in grape, *Vitis vinifera* L. (Salehi et al., 2018), was detected in peanut skin, whole almond seeds, and pistachio kernel (Ballistreri et al., 2009; Ballard et al., 2010; Xie and Bolling, 2014; Čolić et al., 2017; Bodoira and Maestri, 2020). Moreover, various types of stilbene derivatives were also found. Two prenylated resveratrols, arachidin I and II, were found in the peanut kernel (Bisby, 1994). In whole almond seed, the glycosylated resveratrol named polydatin is the most prominently detected stilbenoid along with small amounts of a methylated and two hydroxylated resveratrols called pterostilbene, piceatannol, and oxyresveratrol, respectively (Xie and Bolling, 2014).

### Lignans and Coumarins

Although lignans are not well-investigated in nut plant species, several major plant lignans and their hydroxylated derivatives have been reported in the nut species that we are reviewing in this article, such as lariciresinol, matairesinol, secoisolariresinol, cyclolariciresinol, and 7-hydroxymatairesinol, whereas cashew nut contains the highest total lignan contents (Bolling, 2017; Rodríguez-García et al., 2019). The same is true of lignans in nut species; coumarins in seeds of nut plant species have not been characterized well. Some simple and pyrone-substituted coumarin compounds, e.g., aesculin, aesculetin, umbelliferone, and coumestrol, are mostly found in whole almond, Japanese chestnut skin, and Chinese chestnut kernel (Bolling, 2017; Čolić et al., 2017; Tuyen et al., 2017; Chang et al., 2020).

## Recent Updates of the Polyphenolic Biosynthetic Framework in Seeds of Nut Plant Species

**FIGURE 4:**
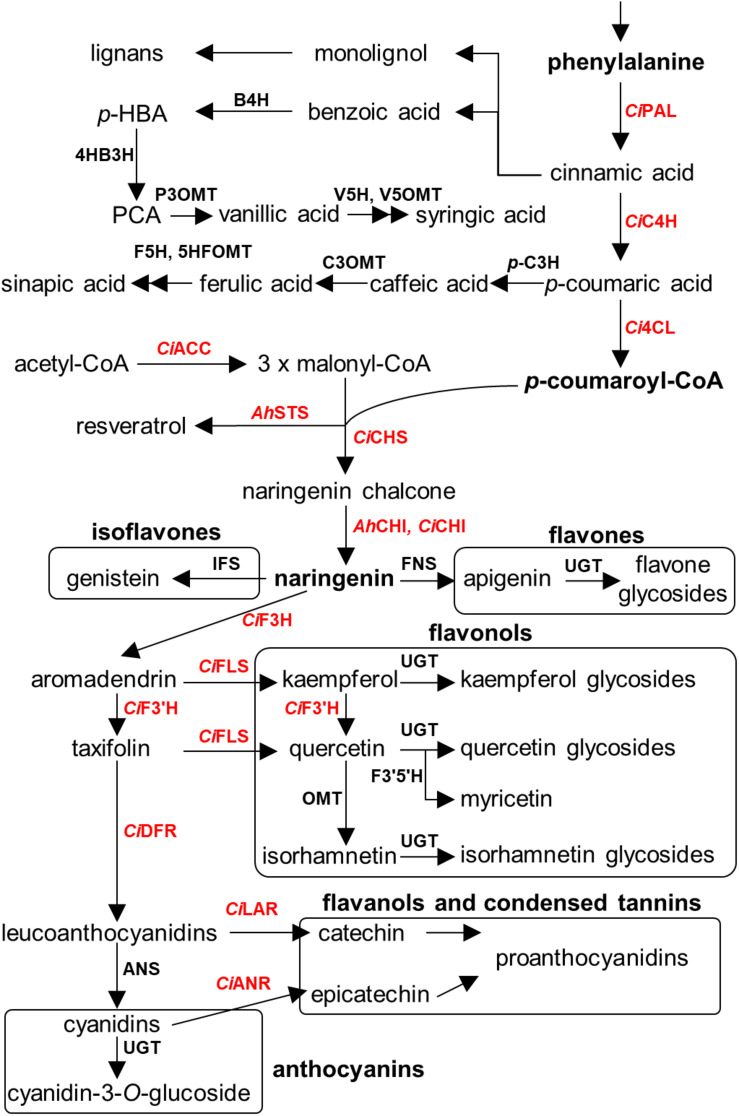
Overview of polyphenolics biosynthesis in major nut plant species. Genes involved in each biosynthetic step are displayed. Characterized genes are shown in red color. Abbreviations used. E4P, erythrose-4-phosphate; PEP, phosphoenolpyruvate; DAHPS, 3-deoxy-D-arabino-heptulosonate 7-phosphate synthase; DHQS, 3-dehydroquinate synthase; DHQ, 3-dehydroquinate; DHD, 3-dehydroquinate dehydratase; DHS, 3-dehydroshikimate; *Jr*GGT, gallate 1-β-glucosyltransferase from walnut; SDH, shikimate dehydrogenase; SK, shikimate kinase; EPSPS, 5-enolpyruvylshikimate 3-phosphate synthase; CS, chorismate synthase; CM, chorismate mutase; HPPD, *p*-hydroxyphenylpyruvate dioxygenase; PAT, prephenate aminotransferase; AD, arogenate dehydratase; *Ci*PAL, phenylalanine ammonia lyase of pecan; B4H, benzoic acid 4-hydroxylase; 4HB3H, 4-hydroxybenzoic acid 3-hydroxylase; P3OMT, protocatechuic acid-3-*O*-methyltransferase; V5H, vanillic acid 5-hydroxylase; V5OMT, vanillic acid 5-*O*-methyltransferase; *Ci*C4H, cinnamic acid-4-hydroxylase from pecan; *p*-C3H, *p*-coumaric acid 3-hydroxylase; C3OMT, caffeic acid 3-*O*-methyltransferase; F5H, ferulic acid 5-hydroxylase; 5HFOMT, 5-hydroxyferulic acid *O*-methyltransferase; *Ci*4CL, 4-coumaroyl-CoA ligase from pecan; *Ci*ACC, acetyl coenzyme A carboxylase from pecan; *Ah*STS, stilbene synthase from peanut; CiCHS, chalcone synthase from pecan; *Ah*CHI, *Ci*CHI, chalcone isomerase from peanut and pecan; IFS, isoflavone synthase; FNS, flavone synthase; *Ci*F3H, flavanone 3-hydroxylase from pecan; *Ci*FLS, flavonol synthase from pecan; *Ci*F3′H, flavonoid 3′-hydroxylase from pecan; *Ci*F3′5′H, flavanoid 3′5′-hydroxylase from pecan; *Ci*DFR, dihydroflavonol reductase from pecan; ANS, anthocyanidin synthase; *Ci*LAR, leucoanthocyanidin reductase; *Ci*ANR, anthocyanidin reductase from pecan; and UGT, uridine diphosphate glycosyltransferase.

An overview of the known polyphenolic biosynthetic framework is summarized in Figure 4 (Lushchak and Semchuk, 2012; Cheynier et al., 2013; Anantharaju et al., 2016; Valanciene et al., 2020). Even though the enzymatic genes regarding polyphenolic biosynthesis are elucidated and well characterized in model plants and crop species, such as *Arabidopsis thaliana*, *Zea mays*, and *Camellia sinensis* (Falcone Ferreyra et al., 2012; Jiang et al., 2013), numerous key genes are largely uncharacterized in seeds of nut plant species, probably due to the lower sequence similarity of genes between major model plants and model nut plant species. Currently, the information of biosynthetic genes has been reported only in peanut and pecan. Peanut chalcone isomerase (CHI) types I and II have been identified (Wang et al., 2012; Liu et al., 2015), and stilbene synthase (*AhSTS*), the most vital enzyme for resveratrol biosynthesis, is functionalized (Condori et al., 2009). In the pecan kernel, three chalcone synthases (CHS) were isolated and properly characterized (Zhang et al., 2019b). Moreover, RNA-Seq also provided several other gene candidates of flavonoid biosynthesis, including PAL, C4H, 4CL, CHI, F3H, F3′H, DFR, ANS, LAR, ANR, and MYB transcription factor (Zhang et al., 2019a). However, those candidates have been well identified by Huang and colleagues (Huang et al., 2019). In 2014, Chen et al. (2014) performed the first MYB gene family in peanut investigation and found that AhMYB15 is related to flavonol biosynthesis. Aside from seeds, gallate glucosyltransferase (GGT) I and II are important for β-glucogallin, the intermediate compound for hydrolyzable tannin biosynthesis, which were identified in walnut leaves (Martínez-García et al., 2016). Stilbenoid prenyltransferases named AhR4DT-1 and AhR3′DT-1 involved in prenylated resveratrol production in peanut kernel were explicated in peanut hairy root (Yang et al., 2018). According to current knowledge, it is apparent that the responsible key genes for polyphenolics in the seed of nut plant species remain largely undetermined, especially in the case of decoration enzymatic genes, which are largely responsible for producing chemical structure diversification. Identification of these genes thus represents an important priority for future research.

## Model Nuts: the State of the Art

At present, nut plant species, including peanut (Bertioli et al., 2019), almond (Sánchez-Pérez et al., 2019), pistachio (Zeng et al., 2019), walnut (Martínez-García et al., 2016), and hazelnut (Lucas et al., 2020), have been genome sequenced (Table 1). Furthermore, draft genome sequences of some chestnut cultivars have been currently deposited in the genome database. Such information provides the advantages of those plant species which became “model nut” species for elucidation of physiological and biological functions of key metabolic genes via omics-based approaches. The genome sequence of the cultivated peanut, known as allotetraploids, was elucidated after sequencing its diploid ancestors, *A. duranensis* and *A. ipaensis*, providing helpful hints for peanut domestication (Bertioli et al., 2016). Currently, using peanut genomics data, lipid metabolism (Chen X. et al., 2019) and the genes involved in size and lipid content in seeds, leaf disease resistance, and nitrogen fixing capacity (Zhuang et al., 2019) were investigated and annotated. Importantly, PacBio and chromosome conformation capture (Hi-C) technologies were performed in order to improve data reading quality and a complete peanut genome sequence was lately reported (Bertioli et al., 2019), resulting in a very high-quality genome.

The walnut genome which was first reported in 2016 revealed some of the genes involved in polyphenolic transformations (Martínez-García et al., 2016). The first walnut reference genome was used to generate high-density 700-K single-nucleotide polymorphism (SNP) arrays (Marrano et al., 2019). Importantly, this tool was used to identify gene candidates responsible for flowering process disclosure (Bernard et al., 2020). However, given that the early walnut genome remained at the scaffold level, multi-omics studies for unraveling biological function and regulation were obstructed. For this reason, the *de novo* assembly of the complete walnut genome was attempted by various techniques. The hybridization of walnut species sequencing by single-molecule or PacBio long-read sequencing and optical genome mapping technologies demonstrated a high quality of parental genome sequence (Wu and Gmitter, 2019; Zhu et al., 2019). Recently, nanopore long-read sequencing supplemented with Hi-C technology has been utilized for a high-quality walnut chromosome level genome assembly (Marrano et al., 2020). Illumina sequencing coupled with Hi-C data is also found to provide high-quality genome data and uncover differences (Zhang J. et al., 2020). Almond is also recently described to have a complete genome sequence. The complete genome sequence of almond was initially reported to be coupled with forty-six kilobases of the gene cluster encoding five basic helix–loop–helix (bHLH) transcription factors (Sánchez-Pérez et al., 2019). Fascinatingly, bHLH2 is identified to be involved in amygdalin biosynthesis (Sánchez-Pérez et al., 2019). Alioto and colleagues similarly performed almond genome sequencing and determined transposon elements related to amygdalin biosynthesis and diversification in peach (Alioto et al., 2020). With an ever-increasing number of high-quality nut plant genomes, gene conservation of functional genes is one of the important topics deserving further investigation of plant metabolism. Hazelnut is the most recently reported complete genome sequence by a hybrid sequencing strategy combining short reads, long reads, and proximity ligation methods (Lucas et al., 2020). The European hazelnut (Corylus avellana L. cv. Tombul) was sequenced focusing on gene families encoding hazelnut allergens and the pathogen-resistance locus proteins that are an important for crop improvement in *C. avellana*.

## Biosynthetic Gene Conservation in Nut Genomes

**FIGURE 5:**
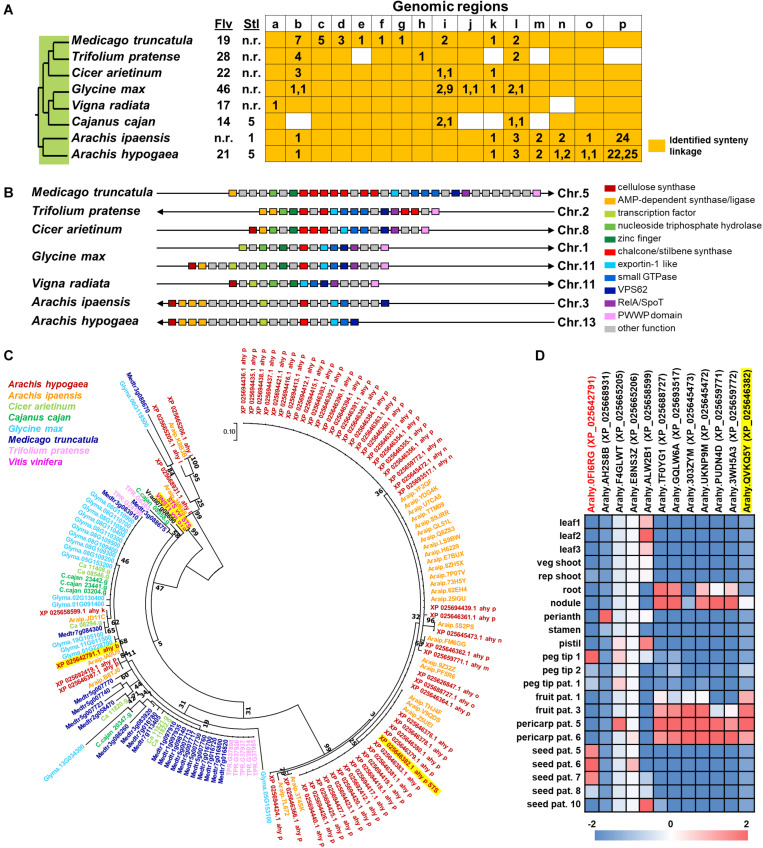
Comparative genomics of CHS and STS in eight legume plant species. **(A)** Genomic synteny analysis of CHS and STS. Gene lists of six legume plant species were retrieved from Plaza database (Dicot 4.5; http://bioinformatics.psb.ugent.be/plaza/). Almond and peanut genome sequences were obtained from the NCBI database. Numbers in each region indicate the number of tandem duplicated genes. Numbers in each region indicate the number of tandem duplicated genes and intra synteny genes. Abbreviations used: Flv, flavonoids; and Stl, stilbenoids. **(B)** Genomic structure of synteny regions b presented in Figure 1A. **(C)** Phylogenetic relationship of CHS and STS located in genome synteny analysis of thirteen plant species. Amino acid sequences were attained from the Plaza database (Dicot 4.5; http://bioinformatics.psb.ugent.be/plaza/) coupled with the NCBI database. The phylogenetic trees were constructed with aligned protein sequences by MEGA7 (Kumar et al., 2016) using the neighbor-joining method with the following parameters: Poisson correction, complete deletion, and bootstrap (1000 replicates, random seed). The protein sequences were aligned by MUSCLE implemented in MEGA. Values on the branches indicate bootstrap support in percentages. The tree with the highest log likelihood (–1287.37) is shown. Colors of circle indicate plant species. **(D)** Gene expression profile of peanut tissues. Each gene is connected with the syntenic region. Gene expression data; part and stage of peanuts are described by previous work of Clevenger and colleagues (Clevenger et al., 2016).

**FIGURE 6:**
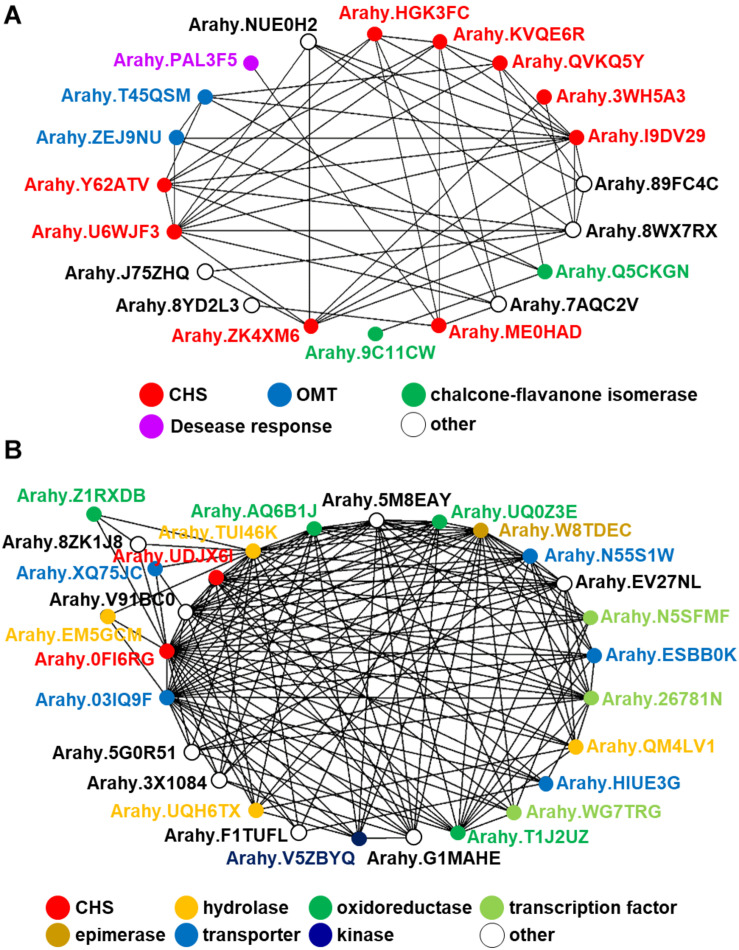
*In silico* co-expression gene network analysis of the peanut STS gene putatively annotated by comparative genomics. **(A)** *AhSTS*, **(B)** *Arahy.0FI6RG*.

In spite of the fact that flavonoids are highly diverse in seeds of nut plant species, the enzymatic gene involved in flavonoid biosynthesis named CHS is frequently mentioned as one of the most conserved key enzymes. In addition, stilbene synthase (STS), the key enzyme of stilbene biosynthesis which resulted in resveratrol as a first product, is discovered as a homolog of CHS since they play a similar function and contain a conserved cysteine residue; hence, STS is described as belonging to the CHS family (Schröder and Schröder, 1990; Lanz et al., 1991). In the nut species presented in this article, *AhSTS1* (arahy.QVKQ5Y, XM_025790597.1, ABY86219.1) in *A. hypogaea* has been characterized (Condori et al., 2009). Both CHS and STS belong to the type III polyketide synthase (PKS) family and have been occurred during functional diversification of PKS (Tohge et al., 2013b). In current informatics research, the phylogenomic synteny network combined with phylogenetic analyses of whole-genomic data of 126 plant species has been developed and focused on the macroevolution of diversification of the PKS family (Naake et al., 2020). In this review, we performed the comparative genomics of CHS and STS between nuts and closely taxonomic-related species by phylogenomic synteny analysis. The total diverged genomic synteny regions of CHS and STS in eight legume plant species are comprised in twenty regions (Figures 5A,B). Three regions (b, k, and l) are commonly found in all studied plant species, whereas m, n, o, and p regions are specific to peanut species. Additionally, the numerous tandem gene duplication is prominently presented in regions b, c, d, I, l, m, n, and p. Even though CHS and STS genes are generally noticed in every mentioned plant species, the flavonoid and/or stilbenoid are still not reported providing a research gap for further metabolite investigation. Based on CHS and STS syntenic regions, a phylogenetic analysis demonstrates the clearly visible separation of region p in wild and cultivated peanut (*Arachis ipaensis* and *A. hypogaea*, respectively), soybean (*Glycine max*), red clover (*Trifolium pretense*), and medicago (*Medicago truncatula*) from the others; it suggests that those uncharacterized genes may function as CHSs (Figure 5C). Interestingly, two characterized grapevine STSs show a separated relationship from *PhSTS* with high bootstrap support. This suggests that these two enzymes evolved from different ancestors may be caused by neo-functionalization of species-specific tandem gene duplications. Nevertheless, a major part of the two displays low bootstrap percentage, which suggests that the CHS and STS phylogeny of the current studied plant species is highly assorted and complex; as such, further investigation is imperative. Apart from genomics investigations, transcriptomic studies are also important for metabolomics studies and crop improvement. The gene expression profiles of various peanut tissues have been revealed (Clevenger et al., 2016). Thus, we attempted to link our synteny analysis with this previously published gene expression data. *phSTS* (XP_025646382.1, ABY86219.1) is expressed in fruits of peanuts. Obviously, XP_025642791 (gene ID. Arahy.0FI6RG) which is pinpointed on region b is mainly expressed during the development of peanut seeds (Figure 5D). Furthermore, a co-expression network analysis of these genes is presented in Figure 6. *phSTS* shows a highly correlated expression with many tandem gene-duplicated CHSs, genes encoding CHI, and *O*-methyltransferase (OMT). On the other hand, Arahy.0FI6RG is correlated with genes related to several types of enzymes, including hydrolases, oxidoreductases, epimerases, and kinases. Moreover, some of these genes are involved in the regulation of transcription factors and transporters. The functions of these candidate genes need to be evaluated experimentally; however, our example of the *in silico* omics-based approach provides insights for future researches into the elucidation of key genes involved in nut polyphenols for metabolomics-assisted breeding approaches aimed at enhancing health-beneficial components.

## Summary and Future Perspective

Nuts are regarded as treasured food crops due to their high contents of potential bioactive components which are able to promote human health benefit. In our summary of the chemical diversity of nut polyphenols, flavonoids are found as the major structurally diversified polyphenols in both aglycone and decorated forms among seeds of nut plant species. With regard to the latter, the glycoside is the main category of polyphenolic derivatives. Diversification of chemical structures results in different effectiveness of biological activities, particularly antioxidants. Although the polyphenolic biosynthetic pathway is widely known, genes of nut plant species encoding enzymes responsible for each step remain uncharacterized. Genome synteny analysis of CHS and STS provides a strategic example for understanding the evolution and conservation of these two enzymes in seeds of nut plant species. Notably, there are several research gaps for nut plant species since much of our knowledge is fragmentary and considerable further investigation is required. Deciphering the multi-omics (genomics, transcriptomics, proteomics, and metabolomics) of nut plant species will provide fundamental data for their physiological function and potential for crop improvement, including increasing crop yield, stress, and disease tolerance, as well as enhance the production of human health beneficial specialized metabolites.

## Author Contributions

CA and TT wrote the manuscript. CA, MW, and TT conceived, designed, and conceptualized the outline of the manuscript. TS, CA, and TN performed comparative genome analysis. VS, SB, MW, AF, and TT supervised and edited the manuscript. All authors have read and approved the final manuscript.

## Conflict of Interest

The authors declare that the research was conducted in the absence of any commercial or financial relationships that could be construed as a potential conflict of interest.
